# Antiepileptogenic Effect of Subchronic Palmitoylethanolamide Treatment in a Mouse Model of Acute Epilepsy

**DOI:** 10.3389/fnmol.2018.00067

**Published:** 2018-03-14

**Authors:** Julia M. Post, Sebastian Loch, Raissa Lerner, Floortje Remmers, Ermelinda Lomazzo, Beat Lutz, Laura Bindila

**Affiliations:** Institute of Physiological Chemistry, University Medical Center of the Johannes Gutenberg University of Mainz, Mainz, Germany

**Keywords:** palmitoylethanolamide, LC-MRM, endocannabinoids, eicosanoids, lipidomics, epilepsy, FAAH inhibitors, antiepileptic drugs

## Abstract

Research on the antiepileptic effects of (endo-)cannabinoids has remarkably progressed in the years following the discovery of fundamental role of the endocannabinoid (eCB) system in controlling neural excitability. Moreover, an increasing number of well-documented cases of epilepsy patients exhibiting multi-drug resistance report beneficial effects of cannabis use. Pre-clinical and clinical research has increasingly focused on the antiepileptic effectiveness of exogenous administration of cannabinoids and/or pharmacologically induced increase of eCBs such as anandamide (also known as arachidonoylethanolamide [AEA]). Concomitant research has uncovered the contribution of neuroinflammatory processes and peripheral immunity to the onset and progression of epilepsy. Accordingly, modulation of inflammatory pathways such as cyclooxygenase-2 (COX-2) was pursued as alternative therapeutic strategy for epilepsy. Palmitoylethanolamide (PEA) is an endogenous fatty acid amide related to the centrally and peripherally present eCB AEA, and is a naturally occurring nutrient that has long been recognized for its analgesic and anti-inflammatory properties. Neuroprotective and anti-hyperalgesic properties of PEA were evidenced in neurodegenerative diseases, and antiepileptic effects in pentylenetetrazol (PTZ), maximal electroshock (MES) and amygdaloid kindling models of epileptic seizures. Moreover, numerous clinical trials in chronic pain revealed that PEA treatment is devoid of addiction potential, dose limiting side effects and psychoactive effects, rendering PEA an appealing candidate as antiepileptic compound or adjuvant. In the present study, we aimed at assessing antiepileptic properties of PEA in a mouse model of acute epileptic seizures induced by systemic administration of kainic acid (KA). KA-induced epilepsy in rodents is assumed to resemble to different extents human temporal lobe epilepsy (TLE) depending on the route of KA administration; intracerebral (i.c.) injection was recently shown to most closely mimic human TLE, while systemic KA administration causes more widespread pathological damage, both in brain and periphery. To explore the potential of PEA to exert therapeutic effects both in brain and periphery, acute and subchronic administration of PEA by intraperitoneal (i.p.) injection was assessed on mice with systemically administered KA. Specifically, we investigated: (i) neuroprotective and anticonvulsant properties of acute and subchronic PEA treatment in KA-induced seizure models, and (ii) temporal dynamics of eCB and eicosanoid (eiC) levels in hippocampus and plasma over 180 min post seizure induction in PEA-treated and non-treated KA-injected mice vs. vehicle injected mice. Finally, we compared the systemic PEA treatment with, and in combination with, pharmacological blockade of fatty acid amide hydrolase (FAAH) in brain and periphery, in terms of anticonvulsant properties and modulation of eCBs and eiCs. Here, we demonstrate that subchronic administration of PEA significantly alleviates seizure intensity, promotes neuroprotection and induces modulation of the plasma and hippocampal eCB and eiC levels in systemic KA-injected mice.

## Introduction

Epilepsy is one of the most common neurological disorders worldwide with severe impact on the life quality of patients and often leading to long-term cognitive impairments (Xu et al., 2013). Although numerous antiepileptic drugs (AEDs) are currently available, they mainly target symptoms rather than underlying molecular mechanisms and often cause massive side effects substantially limiting their therapeutic use (Cully, 2014; Eisenstein, 2014; Savage, 2014). Moreover, a high variability in pharmaco-sensitivity among patients and high incidence of multi-drug resistance in patients pose tremendous challenges in therapy management and development of efficient antiepileptic therapies (Katona, 2015). Multiple causes of epileptogenesis such as head trauma, genetic and metabolic factors, and infections, combined with the diversity of epileptic manifestation and types, and yet unclarified mechanism of epileptogenesis challenge the development of effective antiepileptic therapies (Narain, 2014; Amini et al., 2015). In this context, identification of molecular causes of and/or correlates with epilepsy in various animal models is essential to discover new drug targets and markers for follow-up monitoring.

The intrinsic role of the endocannabinoid (eCB) system to control neuronal network excitability has shaped the focus of pharmacological approaches in epilepsy on cannabinoid-based therapies (Citraro et al., 2013; Monory et al., 2015). Cannabis has been long used as an effective antiepileptic compound. However, a large variability in patient response, psychoactive effects, possible long-term side effects in young patients, as well as the unpredictable risk of cannabinoid-receptor 1 (CB1) desensitization subsequently reducing antiepileptic effects or even aggravating seizures, remain main concerns for its clinical use (Lutz, 2004; Szaflarski and Bebin, 2014; Blair et al., 2015; Katona, 2015; Mechoulam, 2017).

Alternative approaches to modulate the hyperexcitability with seizure via activation of CB1 and its ligands, include the pharmacological blockade of enzymes involved in the degradation of endogenous neuroprotectants; anandamide (also known as arachidonoylethanolamide [AEA]), palmitoylethanolamide (PEA), and oleoylethanolamide (OEA). Several inhibitors of the fatty acid amide hydrolase (FAAH) which degrades AEA, PEA and OEA were developed and assessed for their anticonvulsant properties (Vilela et al., 2013, 2014; Mikheeva et al., 2017). Although effective anticonvulsant properties were evidenced for many FAAH inhibitors, their biphasic effect has to be carefully considered to prevent pro-convulsant effects (Di Marzo et al., 1994). This is likely the source of inconsistencies among various reports on the use of FAAH inhibitors (Vilela et al., 2013; Rivera et al., 2015). Exogenous administration of AEA has similar biphasic effects, in a dose-dependent manner, in epileptic seizures.

PEA, the fatty acid amide analog of AEA, has become the focus of increasing attention due to its long recognized anti-inflammatory and neuroprotective properties (Lambert et al., 2001; Conti et al., 2002; Mattace Raso et al., 2014). It has been also shown to exert antiepileptic effects in three epilepsy models (Lambert et al., 2001; Sheerin et al., 2004). The mechanism of action of PEA in brain and periphery is still not clarified (Iannotti et al., 2016). Although initially believed that PEA is a cannabinoid CB2 receptor agonist, consensus has emerged that PEA acts through activation of peroxisome proliferator-activated receptor α (PPARα), which is a ubiquitous transcription factor in the periphery (LoVerme et al., 2006; Hansen, 2010). PPARs regulate gene networks by switching off signaling cascades involved in gene transcription leading to the release of pro-inflammatory mediators (Verme et al., 2005; D’Agostino et al., 2007, 2009). PPARα is also present in hippocampus, corpus striatum, spinal cord and frontal cortex, opening new venues for research on the mechanism of PEA-mediated neuroprotection and neuroinflammation in the central nervous system (CNS; Moreno et al., 2004). Through activation of PPARα, PEA activate several different receptors including vanilloid-receptor, and ion channels involved in neuronal firing (Hansen, 2010). The mechanism by which systemically administered PEA mediates neuroprotective and anti-inflammatory effects in CNS has not been yet elucidated, and is of emerging interest. It has been recently demonstrated that at least the anti-inflammatory effects of PEA are mediated by a cross-talk between glia cells and mast cells in periphery and brain, whereby PEA blocks the activation of mast cells in the brain and periphery, thus inhibiting inflammatory signaling pathways involved in the periphery-brain cross-talk (Skaper et al., 2013, 2014).

Neuroinflammatory processes resulting from excitotoxicity in epileptic seizures potentiate the inflammatory responses, mediated by cyclooxygenase-2 (COX-2) derived eicosanoids (eiCs) such as prostaglandin E_2_ (PGE_2_) and prostaglandin D_2_ (PGD_2_), and accelerate neuronal hyperexcitability, seizure extent and reoccurrence (Serrano et al., 2011; Vezzani et al., 2013; Barker-Haliski et al., 2017; Terrone et al., 2017). Targeting brain inflammation signaling pathways constitutes a complementary approach or adjuvant therapy to AEDs, particularly in patients with refractory epilepsy (Dey et al., 2016).

In recent years, evidences accumulated also on the role of systemic inflammation and/or peripheral immunity activation in rendering propensity to seizure occurrence and progression. Overexpression of pro-inflammatory mediators, e.g., cytokines, prostaglandins, nitric oxide signaling, and/or endogenous opioids in hippocampal tissue was evidenced to follow peripheral inflammation with subsequently increased neuronal excitability in animal models underscoring the occurrence of brain immune system communication (Riazi et al., 2010; Murta et al., 2015). Peripheral inflammation induced by lipopolisacharides led to increased neuroinflammatory processes, oxidative stress, and seizure susceptibility in a rat brain of kainic acid (KA)-induced excitotoxicity. This effect was mainly reversible by COX-2 inhibitor mediated neuroprotection (Ho et al., 2015). Peripheral anti-inflammatory treatment with anti-interleukin (IL)-1β has been shown to reduce seizure severity in a pilocarpine model (Marchi et al., 2009). In human studies, a relation between inflammatory processes, immunity and seizure susceptibility, occurrence and intensity were evidenced for patients suffering from different epilepsy types including temporal lobe epilepsy (TLE; Gupta and Appleton, 2005; Buzatu et al., 2009; Hancock et al., 2013; van den Munckhof et al., 2015). Interleukin 6 (IL-6) levels in cerebrospinal fluid (CSF) and plasma from epilepsy patients were significantly increased 24 h post tonic-clonic seizures, and in patients with TLE serum levels of IL-6 and IL-β1 remain upregulated, indicating a chronic immune mechanism (Silveira et al., 2012; Uludag et al., 2015; de Vries et al., 2016).

We previously evidenced that systemic administration of KA in mice leads to widespread damage in brain and peripheral organs and increased peripheral inflammation at acute seizure state (Lerner et al., 2016), the latter resembling thus a pathological feature of TLE. We specifically aimed at investigating the PEA treatment effectiveness in acute injury phase of the KA-induced excitotoxicity, and therefore we chose a time course of 180 min to reflect this epileptogenesis phase, whereby at 1 h post injection a maximum of seizure activity typically occurs. Therefore, a mouse model of KA-systemic induced epileptic seizure was chosen as suitable to assess the neuroprotective and anti-inflammatory role of PEA, its potential to affect both periphery and brain by inhibiting inflammatory signals, and the anticonvulsive properties. We determined anticonvulsant effects of acute and subchronic administration of PEA by behavioral examination for 180 min post KA injection. Neuroprotective effects of subchronic PEA administration (double injection) were demonstrated in brain sections of PEA-treated vs. non-treated KA-injected animals. Additionally, we evaluated by targeted mass spectrometry-based quantitative profiling the temporal dynamics of eCB- and eiC-levels with epileptic seizures, at 20, 60, 120, and 180 min post KA-seizure induction, and its response upon PEA administration. Anticonvulsive effects of PEA were associated with a modulation of both peripheral and hippocampal levels of eCBs and eiCs. Finally, antiepileptic properties of PEA were compared to those exhibited by systemic pharmacological blockade of FAAH (URB597) and peripheral FAAH blockade by URB937, as well as in combinatorial therapeutic administration.

## Materials and Methods

### Reagents and Chemicals

Calibration standards: arachidonoyl ethanolamide (AEA), 2-arachidonoyl glycerol (2-AG), arachidonic acid (AA), palmitoyl ethanolamide (PEA), 1-arachidonoyl glycerol (1-AG), PGD_2_, prostaglandin E_2_ (PGE_2_), 5(S)-hydroxyeicosatetraenoic acid (5(S)-HETE), 8(S)-hydroxyeicosatetraenoic acid (8(S)-HETE), 12(S)-hydroxyeicosatetraenoic acid (12(S)-HETE), 15(S)-hydroxyeicosatetraenoic acid (15(S)-HETE), 19(S)-hydroxyeicosatetraenoic acid (19(S)-HETE), 20-hydroxyeicosatetraenoic acid (20-HETE), were obtained from BIOMOL Research Laboratories Incorporation (Plymouth Meeting, PA, USA). Internal standards (ISTD): arachidonoyl ethanolamide-d4 (AEA-d4), 2-arachidonoyl glycerol-d_5_ (2-AG-d_5_), arachidonic acid−d_8_ (AA-d_8_), palmitoylethanolamide-d_4_ (PEA-d_4_), 1-arachidonoylglycerol-d_5_ (1-AG-d_5_), prostaglandin D_2_-d_4_ (PGD_2_-d_4_), prostaglandin E_2_-d_9_ (PGE_2_-d_9_), 5(S)-hydroxyeicosatetraenoic acid-d_8_ (5(S)-HETE-d_8_), 12(S)-hydroxyeicosatetraenoic acid-d_8_ (12(S)-HETE-d_8_), 20-hydroxyeicosatetraenoic acid-d_6_ (20-HETE-d6) were obtained from BIOMOL Research Laboratories Incorporation (Plymouth Meeting, PA, USA).

For multiple reaction monitoring (MRM) analysis and lipid extraction, water, acetonitrile (ACN), and formic acid of LC-MS grade were invariably used (Sigma-Aldrich, St. Louis, MO, USA). HPLC grade methyl tert-butyl ether (MTBE) was purchased from Sigma-Aldrich (St. Louis, MO, USA). Ethyl acetate, n-hexane, 2-propanol, and methanol were obtained from Honeywell International Incorporation (Morristown, NJ, USA).

KA was purchased from Abcam plc. (Cambridge, UK). Palmitoylethanolamide (PEA) was obtained from Cayman Chemical (Ann Arbor, MI, USA). FAAH inhibitors, URB597 (3′-(aminocarbonyl)[1,1′-biphenyl]-3-yl)-cyclohexylcarbamate and URB937 (N-cyclohexyl-carbamic acid, 3′-(aminocarbonyl)-6-hydroxy[1,1′-biphenyl]-3-yl ester) was purchased from Cayman Chemical (Ann Arbor, MI, USA). Cremophore and indomethacin were obtained from BIOMOL Research Laboratories Incorporation (Plymouth Meeting, PA, USA).

### Animals, Induction of Excitotoxic Seizures, and Treatments

For this study, C57BL/6N male mice (8–10 weeks of age) were ordered from Janvier Labs (Saint-Berthevin, France). Experimental procedures were carried out in accordance with the European Community’s Council Directive of 22 September 2010 (2010/63EU) and approved by the local animal care committee of the German Rhineland-Palatinate (file reference: 23 177-07/G16-1-075).

**Scheme 1:**
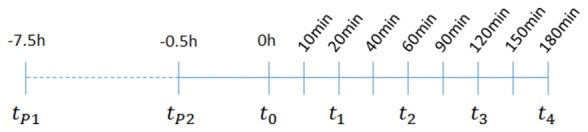
Overview of the time scale for treatment and seizure induction, behavioral scoring, and plasma and hipppocampal sampling: t_1_–t_4_; animal sacrificing, blood and brain tissue sampling; t_p1_–1st palmitoylethanolamide (PEA) administration for subchronic treatment; t_p2_–2nd time point for PEA administration for subchronic treatment, and time point for the acute treatment of PEA, URB597 and URB937; t_0_–time point of kainic acid (KA) injection. For time course analysis of acute KA-induced epilepsy, a duration of 180 min was chosen. At 60 min (t_2_) post KA injection seizures are assumed to reach maximal intensities, therefore this timepoint is referred throughout the study as acute epileptic state, which is preceded by pre- or early acute and followed by late- or after-acute phase.

In order to investigate PEA’s impact on acute epileptic seizures and gain insights into its mechanism of action, mice were treated with freshly prepared KA, as conducted previously (Monory et al., 2006; Lerner et al., 2016) and different pre-treatments were applied (PEA, URB597, URB937) by intraperitoneal injection 7 h and/or 30 min prior to the injection of KA. For an overview see Table 1. All animal experiments were conducted in successive days with intermixed groups. For time course analysis, 24 animals per group were used (receiving a particular treament, vehicle or KA injection according to Table 1), which were subdivided in four groups according to the time point of sacrificing post KA injection. Mice were group-housed (4–5/cage) on a 12-h light/dark schedule. Water and food were available *ad libitum*. The experimenter assessing the behavioral score was blind to the injected agents. Mice were injected intraperitoneally with either KA at a dose of 30 mg/kg body weight or vehicle (0.9% saline, 10 ml/kg body weight; t_0_, Scheme 1). Intraperitoneal pre-treatment injections were conducted with PEA at a dose of 40 mg/kg body weight dissolved in DMSO/chremophor/saline (17:2:1; t_P1_+t_P2_, t_P2_, Scheme 1, Table 1). For combined treatment of PEA with the FAAH-inhibitors URB597 or URB937 (Table 1), the inhibitors were added in the solution (t_P2_, Scheme 1).

**Table 1:** Overview of experimental animal groups.

Group name	Group description	Pre-treatment 7 h prior to KA injection	Pre-treatment 30 min prior to KA injection contains different combinations of PEA/URB937/URB597 dissolved in a mixture of Saline/DMSO/ Cremophore (17:2:1; Vehicle 1)	Injection contains KA dissolved in Saline or Saline for controls (Vehicle 2)	Total nr. *(n)* of animals/group (in each group: *n* = 6 per time point; 20,60,120,180 min)
		t_p2_	t_p2_	t_0_	
**Behavioral scoring and comparative lipidomic profiling in brain & periphery**				
KA	KA-induced seizures	-	Vehicle 1	KA	48^1^
PEA^2^/KA	Subchronic PEA treatment of KA mice	PEA	PEA	KA	24
PEA/KA	Acute PEA treatment of KA mice	-	PEA	KA	48^3^
CTRL 1	Vehicle 2 injection	-	-	Vehicle 2	24^2^
CTRL 2	Vehicle 1 + 2 injection	-	Vehicle 1	Vehicle 2	24^2^
PEA^2^	PEA injection in healthy controls	PEA	PEA	-	24
PEA + URB597/KA	Acute combinatorial PEA + URB597 treatment	-	PEA + URB597	KA	24
URB597/ KA	Acute URB597 treatment	-	URB597	KA	24
PEA + URB937/KA	Acute combinatorial PEA + URB937 treatment	-	PEA + URB937	KA	24
URB937/KA	Acute URB937 treatment	-	URB937	KA	24
**Comparison of neurodegenerative events of PEA treated vs. non-treated epileptic mice and controls via immunohistochemistry and degenerative stains**				
PEA^2^/KA	Subchronic PEA treatment of KA mice	PEA	PEA	KA	4
KA	KA-induced seizures	-	Vehicle 1	KA	4
CTRL	Vehicle 1 + 2 injection		Vehicle 1	Vehicle 2	4

*^1^n = 24 KA-injected mice were compared with acute PEA-treated (PEA/KA) group, n = 24 KA-injected mice were compared with subchronic PEA-treated (PEA^2^/KA) group. ^2^No significant difference in behavior and lipid levels were observed between both control groups (CTRL 1 and CTRL 2). ^3^*n* = 24 acute PEA-treated mice were compared with KA-injected mice, *n* = 24 acute PEA-treated mice were compared with acute URB-treated and combinatorial PEA+URB groups*.

### Behavioral Analysis of Excitotoxic Seizures

Animals were monitored and scored at 10/20/40/60/90/120/ 150/180 min post KA injection according to the following scale: 0—no response; 1—immobility and staring; 2—forelimb and/or tail extension, rigid posture; 3—repetitive movements, head bobbing; 4—rearing and falling; 5—continuous rearing and falling; 6—severe clonic-tonic seizures; 7—death (Monory et al., 2006). Behavior was assessed as a score of 5 in case of continuously rearing and falling for at least three consecutive episodes and without break as a score 6 for generalized convulsive tonic-clonic seizures, as well as for the so-called “popcorn” bouncing activity, characterized by intermediate hyperactive periods of running. Occurrence of the latter type of seizure activity was restricted to short durations (2–4 min) and subsequently caused death. Status epilepticus (SE) was defined as a period of seizure activity, characterized by tonic clonic convulsions without intermediate recovery for >30 min. Mice with SE were assigned the score 6. In the present study, mice were referred to as being acute epileptic with an onset of epileptic motor activity corresponding to seizure score 3 or higher.

**Figure 1:**
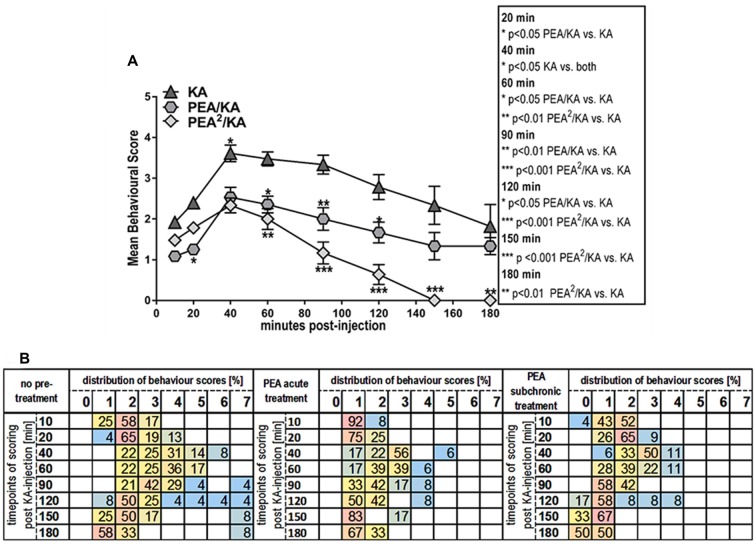
Behavior effects of acute vs. subchronic palmitoylethanolamide (PEA)-treatment of kainic acid (KA)-induced epileptic mice. Behavioral scores were obtained over a time course of 180 min post KA-seizure induction to discriminate effects of acute (PEA/KA) vs. subchronic (PEA^2^/KA) treatment compared to non-treated epileptic mice (KA). **(A)** Repeated measures analysis of variance (ANOVA) revealed significant interaction, thus Tukey’s *post hoc* analysis was performed to determine time-point specific alterations on behavioral level of the different groups. Significant changes of the acute and subchronic PEA treatment vs. non-treated epileptic mice were calculated and assigned as follows: *P < 0.05, **P < 0.01, ***P < 0.001. Error bars indicate standard error of the mean (SEM). At 40 min the significance is indicated for: KA-group vs. both PEA treated groups.** (B)** Behavioral scores given in percentage of the total scores obtained per time point for each experimental groups, presented in a heatmap illustrating comparison of seizure severity and mortality rates between the groups. Group sizes were the following: non pre-treated KA-induced epileptic mice: *n* = 48 at 10 and 20 min; *n* = 36 at 40 and 60 min; *n* = 24 at 90 and 120 min; and *n* = 12 at 150 and 180 min. Acute PEA-treated and subchronically PEA-treated epileptic mice: *n* = 24 at 10, 20 min, *n* = 18 at 40, 60 min, *n* = 12 at 90, 120 min and *n* = 6 at 150, 180 min. KA-seizure induction led to death in 4% of the untreated epileptic mice 90 min post KA injection. According to the applied scoring method, seizure score 7 (death) from the time point they occur is rescored for all the remaining time course. At 120 min mortality rate remained unchanged compared to 90 min, e.g., 4%. From 150 min another 4% of untreated epileptic mice were depicted with score 7 resulting thus in 8% mortality at 150 min which remained unchanged at 180 min. Upon acute PEA treatment no seizure score higher than 5 was obtained, thus compared to untreated epileptic mice, PEA treatment prevents mortality. Subchronic PEA treatment has even more suppressive impact on seizure intensities, with a maximal score of 4 and 50% of the mice with a score of 0 at 180 min.

Animals were scored until they were sacrificed, e.g., 20, 60, 120 or latest 180 min post KA injection, with the last scoring time point immediately prior to sacrificing. Accordingly, number of scores obtained at 10 and 20 min post-KA injection correspond to full size experimental groups. After 40 min, total scores decrease over time in relation to reduction of group size due to sacrificing. Consequently, mice sacrificed at 180 min have a full set of behavior scores over the 3 h time course. To enable appropriate statistical analysis between acute and subchronic PEA treatment, we calculated and assessed averages of all scores obtained within a group/per time point of scoring; 10, 20, 40, 60, 90, 120, 150 and 180 min post KA injection (Figure 1A). The results were analyzed using SPSS Statistics Software for Windows (version 23, IBM, Chicago, IL, USA) or Graphpad (version 7, Prism, San Diego, CA, USA). Repeated-measures analysis of variances (ANOVAs) with experimental group and time as independent variables were used to analyze seizure severity after KA injection. The Greenhouse–Geisser correction was used if the condition of sphericity was not met. Significant group effects were further analyzed using Bonferroni’s *post hoc* analysis for multiple comparisons, whereas significant interactions were further analyzed using simple effects analysis for the effect of group for each time point, also adjusted for multiple testing using Bonferroni correction. The error bars are presented as standard error of the mean (SEM).

To better illustrate distribution of individual scores, i.e., scores higher than 5 and mortality rates, we included a heat map displaying behavior score distribution given in percentage of animals exhibiting a given score from the total number of scored animals (e.g., total number of animals alive at that time point) per experimental group per time point (Figure 1B). In total, 4% of the untreated epileptic mice exhibited SE that eventually led to death for 2%. The 2% of the mice who survived SE, were housed for 5 more days for the purpose of neurodegenerative and immuno-histochemical staining of brain sections that are representative for most severe seizure state, see “Brain and Plasma Sampling” and “Neurodegenerative and Immunohistochemical Staining” sections.

### Brain and Plasma Sampling

At 20/60/120/180 (t_1_–t_4_) minutes after KA injection, mice were shortly anesthetized with isoflurane (Forene R, AbbVie Deutschland GmbH and Co. KG, Wiesbaden, Germany) and sacrificed by decapitation (Nomura et al., 2011; Scheme 1). Brains were isolated and directly frozen on dry ice. Dissection of the brains was carried out according to the protocol described previously (Lerner et al., 2016). All tissue samples were then stored at −80°C until further processing. Blood was collected in ice-cold, 500 μL EDTA plasma extraction tubes (KABE Labortechnik, Nümbrecht-Eisenroth, Germany), containing previously added 10 μM indomethacin to prevent *ex-vivo* COX-2 activity. This collection procedure took 30 s per mouse. Plasma tubes were directly centrifuged at 4°C, 2000 *g* for 10 min, and the resulting upper plasma phase was removed and stored at −80°C without delay.

### Co-extraction and LC/MRM Quantification and Statistical Analysis of eCBs, PEA, AA and eiCs

eCBs and eiCs were co-extracted using a method recently developed by our group. The same sampling, extraction and quantification protocol as previously reported was applied in this study for both plasma and hippocampal eCBs and eiCs investigation (Lerner et al., 2017). The eCB and eiC values were normalized to protein amount and plasma volume for tissue (Bindila and Lutz, 2016) and plasma samples, respectively. In this study we focused on the analysis of AEA, 2-AG, PEA, AA, PGE_2_ and PGD_2_. AEA and 2-AG are eCBs that bind to cannabinoid receptor CB1; PEA is an eCB-like molecule. AA is a precursor for the synthesis of prostaglandins (PGs). We focused our analysis on the PGE_2_ and PGD_2_ due to their prominent involvement in inflammatory processes in general, and arising from KA-induced excitotoxicity, in particular. For the sake of simplicity, we will refer to PGE_2_ and PGD_2_ as eiCs.

Lipid levels were analyzed at each time-point separately by means of univariate ANOVA for experimental group. Significant group effects were further analyzed using Bonferroni’s *post hoc* analysis for multiple comparisons. Statistical significance was defined as *P* < 0.05. Error bars are presented as SEM.

### Perfusion

Eight mice (four mice were subchronically PEA-treated epileptic mice, and four mice were non pre-treated epileptic mice) were kept single housed post KA injection for 5 days. Health conditions of mice were frequently checked, all mice showed normal behavior after acute seizure state (3 h post KA injection) and remained like that even though ongoing neurodegenerative processes are expected. In order to determine effect of subchronic treatment of PEA, Fluoro-Jade C (FJC), silverstaining and immunhistochemical staining were performed. For these purposes, perfusion of the brain is required prior to staining. Therefore, mice were deeply anesthetized using pentobarbital (100 mg/kg) and Buprenorphin (0.1 mg/kg) as narcotic, trans-cardially perfused with phosphate buffered-saline (PBS), followed by perfusion with 4% paraformaldehyde (PFA; ROTH, Carl Roth GmbH+Co KG. Karlsruhe, Deutschland) in PBS. The isolated brains were fixed for 24 h in 4% PFA in PBS solution, treated with 30% sucrose in PBS solution for 48 h and stored at −80°C. Forty micrometer thick free floating coronal brain sections were prepared on a Microm HM560 cryostat (Microm) and stored at 4°C in cryoprotection solution (25% glycerol, 25% ethylene glycol and 50% PBS) until use.

### Neurodegenerative and Immunohistochemical Staining

To enable detection of KA-provoked neurodegeneration and evaluation of PEA’s assumed neuroprotective properties we performed neurodegenerative FJC and silver staining, and additionally, immunohistochemical double staining of neuronal nuclei (NeuN) and caspase-3 (CASP3) activation in brain sections of previously perfused brains. FJC staining was conducted following previously reported protocol (Schmued et al., 2005). Briefly, 40 μm coronal brain sections were collected and mounted, rinsed in ddH_2_O, treated with 1% NaOH (v/v) in 80% EtOH, washed in 70% EtOH and twice in ddH_2_O. Slides were treated with 0.06% KMnO_4_, washed twice in ddH_2_O, and stained with FJC (Millipore, Schwalbach, Germany). Slides were then rinsed twice in ddH_2_O and counterstained with DAPI. The staining was then visualized with a Leica DM5500 fluorescence microscope. For the silver staining, sections were mounted and air dried, then incubated in impregnation solution (saturated LiCO_3_ in ddH_2_O with 10% AgNO_3_ and 25% NH_4_OH) and washed six times in ddH_2_O. Sections were transferred in developer solution (ddH_2_O with 19% formaldehyde (v/v), 14% acetone (v/v), 0.3% hydroquinone (w/v), and 1% Tri-Na-Acetate (w/v)) and washed twice in ddH_2_O. The staining was then visualized with a Leica DM5500 bright field microscope. For immunohistochemistry, 30 μm coronal brain sections of perfused brains were blocked for 90 min in PBS containing 5% normal donkey serum (NDS), 2.5% bovine serum albumin (BSA), and 0.3% Triton X-10 (TX) and then incubated over night with primary antibodies: mouse anti-NeuN (1:1000, Abcam) and rabbit anti-cleaved Casp3 (1:200, Cell Signalling) in PBS containing 1% NDS, 0.1% BSA, and 0.% TX. Next day, secondary antibodies: Goat anti-Mouse IgG, Alexa Fluor 488 (Invitrogen 1:1000) and Goat-anti Rabbit IgG, Alexa Fluor 546 (1:1000 Invitrogen) were applied for 2 h for immunofluorescent staining, visualized with a Leica DM5500 fluorescent microscope and evaluated with a Leica Application Suite Advanced Fluorescence (LAS AF) Software.

In order to comparatively assess the extent of neurodegenerative processes in hippocampal areas from untreated vs. subchronically PEA-treated epileptic mice, image comparison was performed using identical channel settings in terms of exposure time, intensity, gain and threshold. FJC-positive signals were semi-quantitatively analyzed in the hilus region of the hippocampus from PEA-treated vs. untreated epileptic mice. For this purpose, green fluorescent signals were manually counted using ImageJ software in the region of interest (ROI), e.g., hilus, on three consecutive sections from the same animal. The three coronal brain sections were sampled at 200 μm distance across the brain. Brightness and contrast settings were set fixed prior to import of the picture and FJC signals were split from DAPI signals using split channel function. Positive FJC signals were counted using multipoint tool and statistically analyzed via Graphpad prism 7 software.

## Results

### Behavioral Effects of the Systemic Administration of PEA in Mice With KA-Induced Epileptic Seizures

Single injection (acute treatment) of exogenous PEA at 30 min prior to KA injection and double injection of PEA (subchronic administration) at 7 h and 30 min prior to KA injection, rendered significant reduction of seizure intensity (Figure 1A). Indeed, after the acute epileptic state (60 min; Lerner et al., 2016), subchronic PEA administrations exerted significant beneficial effects over the entire 180 min, as compared to single PEA injection, whose effects lasted for 120 min after administration (Figure 1A). Mice undergoing acute PEA treatment (PEA/KA) did not exhibit scores higher than 5, while subchronically treated mice (PEA^2^/KA) not higher than 4 (Figure 1B). In contrast, 8% of untreated epileptic mice (KA) died, 4% exhibited SE (assigned as score 6; Figure 1B) that eventually led to death for 2%. It is of note that normal behavior (score 0) was observed only for mice with subchronic PEA treatment at 10 min post KA administration and more prominent, at late acute seizure state, 120–180 min.

### Temporal Dynamics of eCB and eiC Levels in Hippocampus and Plasma of Mice With KA-Induced Epilepsy and Upon Subchronic PEA Treatment

#### Hippocampus

**Figure 2:**
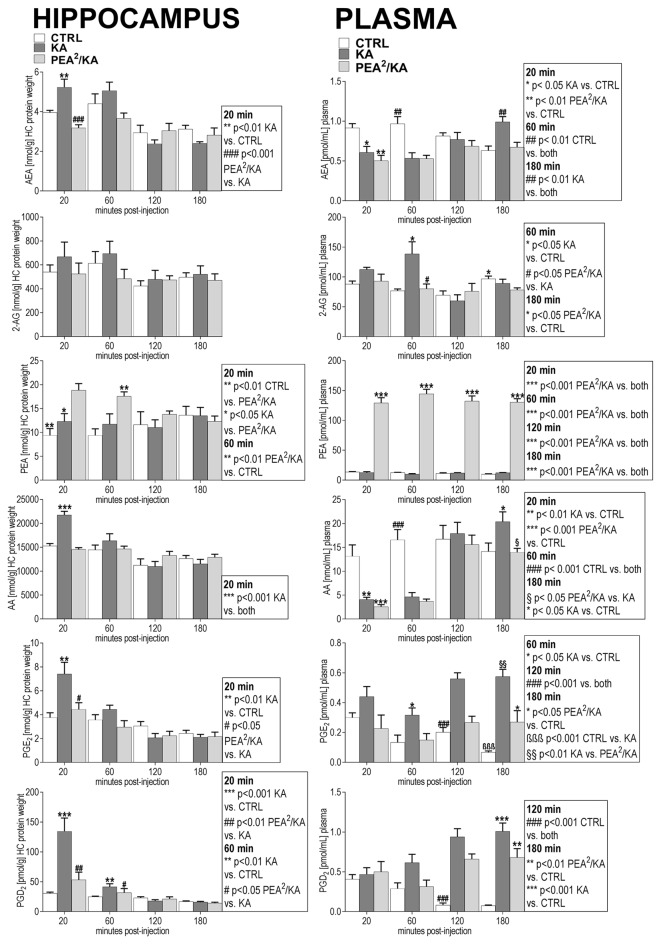
Impact of PEA treatment on temporal dynamics of hippocampal and plasma lipid levels in KA-induced epilepsy mouse model. Anandamide (also known as arachidonoylethanolamide [AEA]), 2-arachidonoylglycerol (2-AG), PEA, arachidonic acid (AA), prostaglandin E2 (PGE_2_) and prostaglandin D2 (PGD_2_) were comparatively analyzed at 20, 60, 120, 180 min post KA injection, respectively, in KA-injected animals (KA) vs. vehicle injected (Ctrl) and subchronically PEA-treated KA injected animals (PEA^2^/KA). Error bars indicate SEM and asterisks in the figures indicate significant differences, *P < 0.05, **P < 0.01, ***P < 0.001.

KA-induced epileptic seizure gave rise to an immediate molecular response of AEA, AA, PGE_2_ and PGD_2_ at early acute phase (20 min) post-injection, e.g., all these lipids exhibited a significant increase and returned then to basal level (Figure 2). Subchronic administration of exogenous PEA produced significant effects on the seizure-induced hippocampal increase of AEA, AA, PGE_2_ and PGD_2_. The immediate (20 min) increase of AEA and AA with seizure is reversed to basal by subchronic PEA treatment. Moreover, PEA significantly alleviated the KA-induced increase of the inflammatory markers PGE_2_ and PGD_2_ to basal level (Figure 2). The hippocampal level of PEA was significantly augmented at 20 and 60 min upon subchronic administration of PEA (Figure 2), but not with acute PEA treatment (Supplementary Figure S1).

Both, acute and subchronic treatment with PEA of KA-mice modulated the eCB and eiC hippocampal levels. However, the acute PEA treatment alleviated to a lower extent the KA-induced eCB and eiC changes compared to subchronic treatment (Supplementary Figure S1).

#### Plasma

KA-induced epileptic seizures gave rise to a different temporal change of eCB and eiC levels in plasma in comparison with hippocampus. At 20 min, a significant decrease of the plasma levels of AEA and AA occurred, which was maintained also at the acute seizure state (60 min). Their levels returned to basal at 120 min followed by a significant elevation at 180 min post-KA injection (Figure 2). 2-AG levels were significantly elevated only at acute seizure state (60 min) compared to controls (Figure 2). Compared to hippocampus (Figure 2), in plasma a temporal shift in the increase of the inflammatory markers PGE_2_ and PGD_2_ in KA-injected mice occur: PGE_2_ increased significantly at the acute state and was maintained highly elevated up to 180 min post-injection, whereas PGD_2_ increased in the late-acute seizure phase at 120 min and remained elevated at 180 min after KA injection. Plasma PEA levels remained unaffected by epileptic seizures throughout the 180 min-lasting time course analysis.

Subchronic PEA treatment did not restore AEA and AA alterations in the immediate (20 min) and acute phase (60 min) of seizure, but it exerted significant effects in the late-phase of seizure progression occurring at 180 min post KA injection. The increase of 2-AG levels observed in the acute seizure phase (Figure 2) was normalized to basal upon subchronic PEA treatment. At late seizure-phase, 180 min post KA-injection, subchronic PEA administration decreased the 2-AG level compared to controls. Subchronic PEA treatment reversed to basal level the increase of PGE_2_ in the acute phase of epilepsy (60 min), while it induced a substantial alleviation but not complete recovery of the increased PGE_2_ levels at 120 min and 180 min post-seizure induction. The late-phase (120 min and 180 min) increase of PGD_2_ with seizures was also attenuated but not to basal level by subchronic PEA treatment. Subchronic PEA dose more effectively alleviated the modulation of plasma eiCs with seizure compared to acute PEA treatment (Supplementary Figure S2).

Subchronic PEA treatment in KA-injected mice led to a significant increase of PEA levels in plasma over the entire time course (180 min) due to accumulation of exogenous PEA, which was peripherally administered. Upon single dose PEA administration, plasma PEA levels were significantly higher than in controls and non-treated KA-injected mice up to 120 min, and were then restored to basal at 180 min post KA injection (Supplementary Figure S2).

Both, hippocampus and plasma exhibited alterations with epilepsy of the eiCs deriving from the COX-2 pathway. The LOX pathway-derived metabolites of AA, 5(S)-HETE, 8(S)-HETE, 12(S)-HETE, 15(S)-HETE, 19(S)-HETE and 20-HETE, remained unaltered with epilepsy and upon PEA treatment (data not shown), indicating a specific role of COX-2 mediated inflammation induced by epilepsy. These results concur with other findings reporting that the COX-2 mediated inflammation process, which result from KA-induced excitotoxicity can potentiate or sustain seizure intensity and/or promote re-occurrence of seizures (Serrano et al., 2011; Vezzani et al., 2013; Rojas et al., 2014).

### PEA Effect in Controls

Intraperitoneal subchronic administration of PEA in controls and vehicle injection in controls was performed to understand whether and how PEA modulates the hippocampal and plasma eCB and eiC levels under healthy physiological conditions. In plasma, in the absence of epilepsy, subchronic PEA treatment significantly increased the AEA levels and decreased 2-AG over the entire time course (Supplementary Figure S3). AA levels in plasma were significantly decreased compared to vehicle-injected mice up to 1 h. Inflammation markers, PGE_2_ at 20 and 120 min, and PGD_2_ at 20 min post KA injection, respectively, were also significantly decreased upon subchronic PEA administration in controls (Supplementary Figure S3). As expected, plasma PEA levels were significantly increased upon exogenous PEA administration in controls over the entire time-course, e.g., 180 min post-injection. Subchronic administration of PEA in controls significantly augmented in hippocampus only the levels of PEA and AA (Supplementary Figure S4), which suggests an up-take of exogenous PEA in the brain.

### PEA Effect on Neurodegeneration

**Figure 3:**
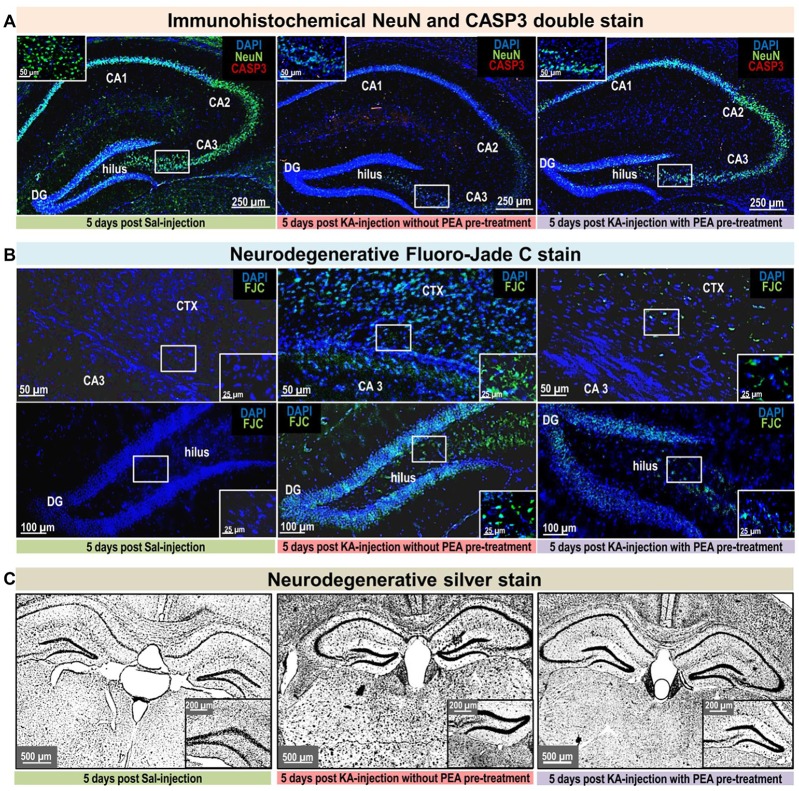
Neurodegenerative and immunohistological staining 5 days post KA-seizure induction. To unravel PEA’s neuroprotective potential neurodegenerative and immunostainings were performed on brain sections sampled 5 days post KA-induced epileptic seizures in mice without subchronic PEA treatment (red labeled middle column), with subchronic PEA treatment (purple labeled right column) and of saline injected control mice (green labeled left column). For these animals, behavioral scores were obtained over a time course of 180 min (post KA/Saline-injection) analog to the procedure described for the other experimental groups of this study (see “Materials and Methods” section). No behavioral changes were observed for control animals (Panels **A–C**; left image, respectively). Untreated epileptic mice exhibited maximal seizure scores from 5 (continuously rearing and falling) for 15 min 1 h post KA injection (Panel **B** upper middle image) to 6 (Status epilepticus (SE)) for 70 min 1 h post KA injection (Panel **A**; middle image, Panel **B**; lower middle image and Panel **C**; middle image). Subchronic PEA-treated epileptic mice exhibited a maximal seizure score of 4 (rearing and falling) for 15 min post KA injection (Panels **A–C**; right image, respectively). Brain sections were compared regarding neurodegeneration by **(A)**. Neuronal nuclei protein (NeuN) staining (green signal) and caspase-3 (CASP3) activation (red signal), **(B)** Fluoro-Jade C (FJC) staining (green signal), and **(C)** Silver staining. KA- induced SE causes massive loss of NeuN signal, predominantly in the CA1, CA3 and hilus region of hippocampus, accompanied by apoptotic events indicated by CASP3 signal (Panel **A**; middle image) compared to control (Panel **A**; left image). Subchronic PEA-treatment (Panel **A**; right image) notably preserves NeuN signal, whereas CASP3 signal is barely detectable. FJC staining indicates neurodegeneration predominantly in cortical layer adjacent to CA3 region of the hippocampus in untreated epileptic mice with a maximal score of 4 (Panel **B**; upper middle image). In untreated epileptic mice with SE (Panel **B**, lower middle image) a widely spread FJC-signal throughout DG and hilus region is observed. Brain sections from subchronically PEA-treated mice display comparatively weak occurrence of neurodegenerative events (Panel **B**; upper and lower right image), while sections from control mice do not show FJC-signal (Panel **B**; upper and lower left image). Silver stained brain sections of untreated epileptic mice (Panel **C**; middle image) indicate widespread neurodegenerative effect of KA-induced SE and comparatively only moderate effect on brain sections from PEA-treated mice (Panel **C**; right image) in relation to brain section from healthy control mice (Panel **C**; left side).

Immunohistochemical double staining of NeuN and CASP3 (Figure 3A), as well as neurodegenerative FJC- (Figure 3B) and silver (Figure 3C) staining on brain sections sampled 5 days post KA-seizure induction revealed neurodegenerative processes in mice without subchronic PEA treatment compared to saline injected control mice.

KA-induced SE causes massive loss of NeuN signal and apoptosis in untreated epileptic mice, compared to controls, while subchronic PEA treatment notably preserves NeuN signal without showing indication of apoptotic events. Distribution of FJC- stained neurodegeneration correlates with maximal seizure intensities, that were observed within 180 min post KA injection: SE (score 6) causes widespread FJC-signal in hippocampal areas (Figure 3B; lower middle image), while seizure score of 5 results in less spread FJC-signal, predominately located adjacent to CA3 region (Figure 3B; upper middle image). In comparison, upon subchronic PEA treatment a behavior score of 4 was obtained and FJC-signal occurs to a lower extent (Figure 3B; upper and lower image).

Semiquantitative comparison of positive FJC signals in the hilus region 5 days post KA injection revealed significantly reduced (**P < 0.0016 KA vs. PEA^2^/KA) levels of FJC-sensitive neurodegeneration upon subchronic PEA treatment (the treated mouse had a max. behavioral score of 4 within the 180 min time-course) compared to FJC-signals in the hilus regions obtained 5 days post KA-induced SE (Supplementary Figure S5).

Silver staining indicates widespread neurodegenerative effects provoked by KA-induced SE (Figure 3C, middle image), while a moderate extent of neurodegeneration was observed on brain section from mice with subchronic PEA treatment (Figure 3C; right image) in relation to brain section from healthy control mice (Figure 3C; left side).

### Comparison of PEA, URB597, URB937, and Combinatorial PEA With URB597 and URB937 Treatments in KA-Injected Mice

Pharmacological blockade of FAAH has been proposed as an effective anticonvulsant therapy, due to the increases of endogenous levels of the AEA and PEA, whose function as endogenous neuroprotectants is well established. URB597 is an FAAH inhibitor, which acts systemically by irreversible FAAH blockade and has a biphasic effect as an anti- or pro-convulsant agent in a dose-dependent manner. Therefore, we aimed at comparing the effectiveness of exogenous PEA and URB597 as anticonvulsant agents and at gaining insights into how the two therapeutic approaches modulate eCBs and eiCs in brain and periphery. Moreover, by using specific blockade of peripheral FAAH by URB937 we aimed at gaining some insights into the contribution of peripheral increase of AEA and PEA levels to seizure alleviation, hence anticonvulsant effects. Another rationale for this comparison is that PEA reportedly potentiates the AED efficiency (Citraro et al., 2016) and the effect of AEA on the cannabinoid receptor (CBR) and/or vanilloid receptor 1 (TRPV1; Ben-Shabat et al., 1998; De Petrocellis et al., 2001; Costa et al., 2008). We therefore, performed a time course study of the effects of URB597, URB937 and each in combination with exogenous PEA administration on the modulation of AEA and PEA, as well as related eCB and eiCs in brain and plasma, and of the anticonvulsant properties in epileptic mice in comparison with PEA-treated epileptic mice.

### Anticonvulsant Properties of URB597, PEA and Combined PEA/URB597 in KA-Injected Mice

**Figure 4:**
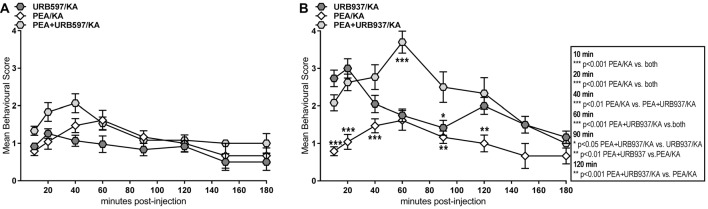
Behavior effects of URB597 and URB937 vs. PEA treatment of KA-induced epileptic mice. Behavioral scores were obtained over a time course of 180 min post KA-seizure induction in **(A)** URB597-treated (URB597/KA), and combined, PEA- and URB597-treated (PEA/URB597/KA) vs. acute PEA-treated (PEA/KA) mice, as well as in **(B)** URB937-treated (URB937/KA), and combined, PEA- and URB937-treated (PEA+URB937/KA) vs. acute PEA-treated (PEA/KA) mice. Error bars indicate SEM and asterisks in the figures indicate significant differences, *P < 0.05, **P < 0.01, ***P < 0.001.

Behavioral analysis of seizure intensity over 180 min post-KA injection in mice treated with PEA, URB597, and combination of PEA and URB597 showed no significant differences between the three groups (Figure 4A). This indicates that PEA-induced anticonvulsion is similarly effective as for URB597 and that co-administration with URB597 does not induce any cumulative therapeutic effect due to drug interaction (Figure 4A).

### Anticonvulsant Properties of URB937, PEA and Combined PEA/URB937 in KA-Injected Mice

Pharmacological blockade of peripheral FAAH was less effective than PEA treatment in maintaining anticonvulsive effects over the entire time course. This is evident in the pre- and late-acute seizure phase, where URB937-treated mice showed significantly higher convulsion intensity compared to those animals treated with PEA only (Figure 4B). Moreover, the combination of PEA/URB937 fails to be effective in the acute (60 min) and late-acute seizure phase (60–120 min), suggesting some mutually exclusive effects of these combined treatments in the acute seizure state. This also suggests that, upon FAAH blockade, the pharmacological increase of AEA and PEA must occur also in the brain in order to exert an effective therapeutic action for seizure prevention.

### Modulation of the Peripheral and Hippocampal eCBs and eiCs by URB597 in KA-Injected Mice

**Figure 5:**
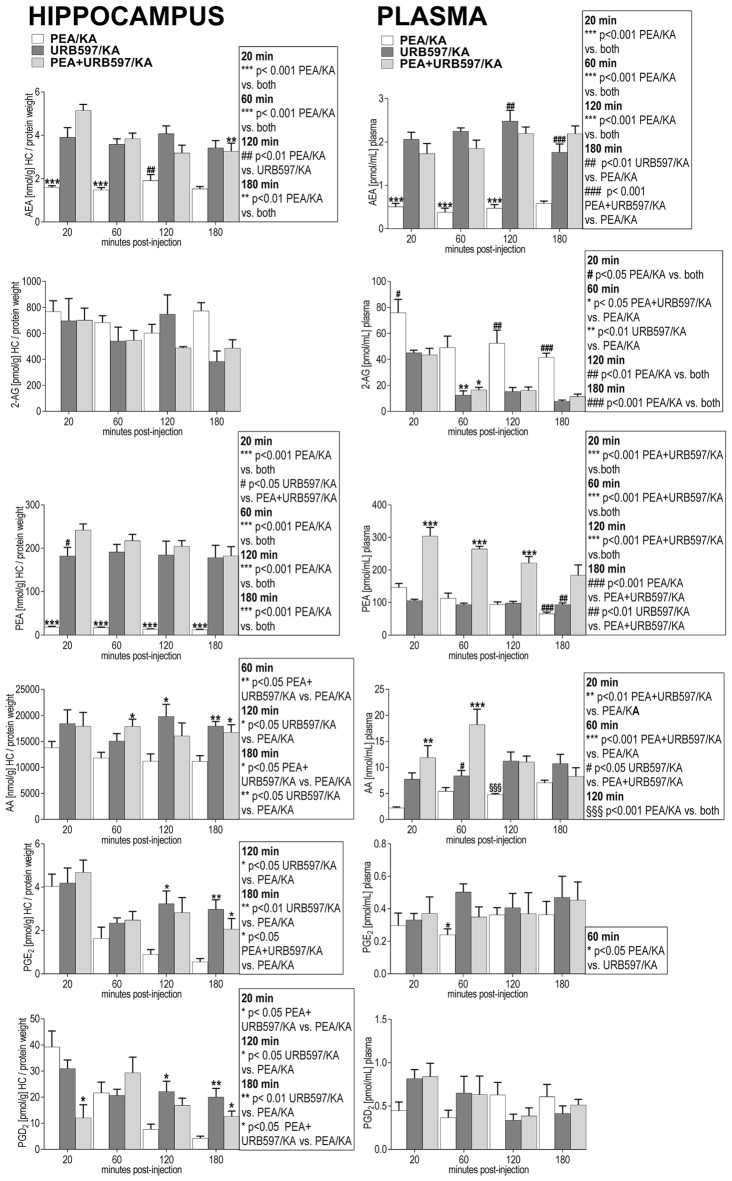
Impact of URB597 treatment on temporal dynamics of hippocampal and plasma lipid levels in KA induced epilepsy mouse model. AEA, 2-AG, PEA, AA, PGE_2_ and PGD_2_ were comparatively analyzed in URB597-treated mice (URB597/KA) vs. combined, PEA- and URB597-treated (PEA+URB597/KA), and acute PEA-treated (PEA/KA) mice at 20, 60, 120, 180 min post KA injection, respectively. Error bars indicate SEM and asterisks in the figures indicate significant differences, *P < 0.05, **P < 0.01, ***P < 0.001.

In hippocampus, administration of URB597 boosts the production of endogenous AEA and PEA, which occurs already at 20 min post seizure induction and is maintained at a significant high level over the 180 min time course (Figure 5). Combined administration of PEA/URB597 in KA-injected mice had similar effects as URB597 treatment on the hippocampal AEA and PEA levels, indicating that no cumulative effects of exogenous PEA administration and blockage of PEA and AEA degradation by URB597 occur (Figure 5).

URB597 administration maintains in hippocampus at late-acute seizure phase (120–180 min) a higher level of inflammation markers, PGE_2_ and PGD_2_ compared to PEA administration only. This molecular feature occurs also when PEA and URB597 are co-administered, but is less significant compared to URB597 treatment alone (Figure 5). Similar effects are observed for AA at and after acute seizure state (60–180 min) upon URB597 and URB597/PEA treatment compared to PEA treatment only.

In periphery, URB597-mediated inhibition of AEA degradation (Figure 5) was maintained over the entire time course also when co-administered with PEA, rendering in periphery constant, significantly higher levels of AEA compared to PEA-treated mice. Exogenous administration of PEA and inhibition of endogenous PEA degradation by URB597 had similar effects on the PEA level in plasma. The combined treatment with URB597 and PEA, however, rendered a cumulative effect on the plasma PEA level over the entire time course indicated by the significantly higher levels of plasma PEA compared to those obtained by URB597 or PEA treatment alone (Figure 5). URB597 and PEA/URB597 treatments have a pronounced effect on plasma 2-AG over the entire time course maintaining it at significantly lower levels compared to PEA treatment. Conversely, AA levels are significantly augmented for up to 120 min upon URB597 and URB597/PEA compared to PEA treatment. PEA, URB597 and URB597/PEA treatments modulate the inflammation markers PGE_2_ and PGD_2_ in a similar manner and extent, except for acute phase where the PGE_2_ level is maintained higher upon URB597 compared to PEA treatment (Figure 5).

### Modulation of the Peripheral and Hippocampal eCBs and eiCs by URB937 in KA-Injected Mice

**Figure 6:**
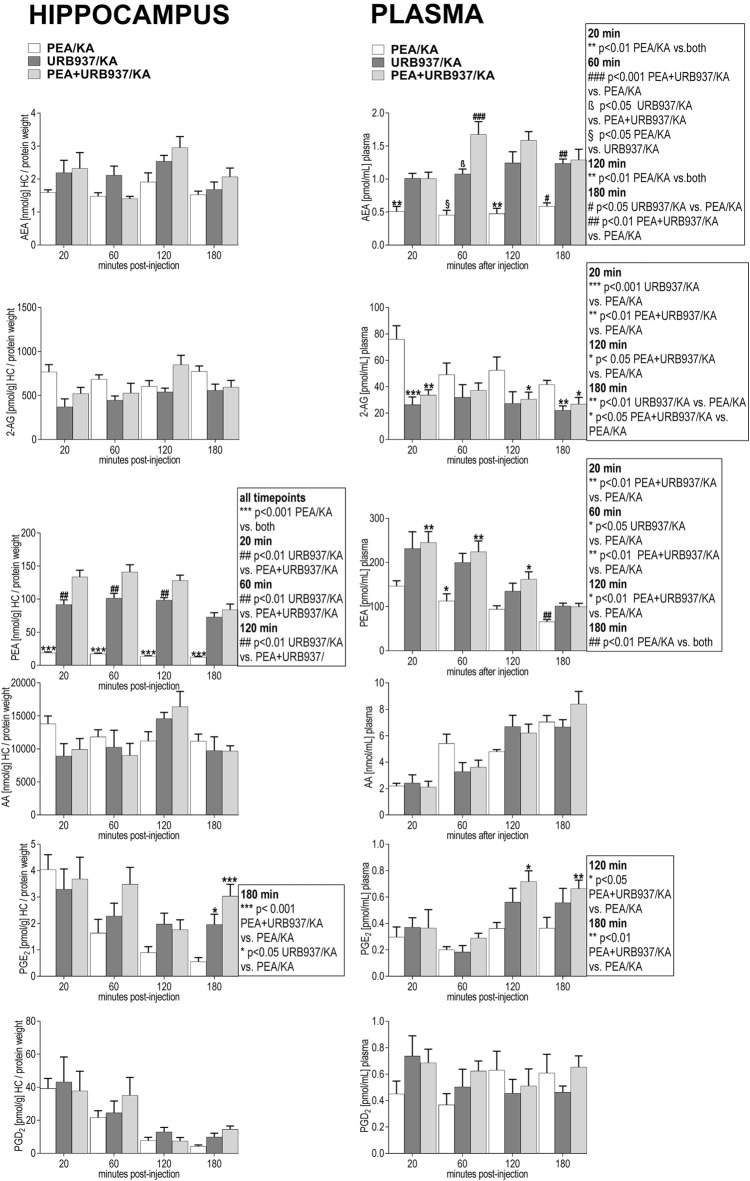
Impact of URB937 treatment on temporal dynamics of hippocampal and plasma lipid levels in KA induced epilepsy mouse model. AEA, 2-AG, PEA, AA, PGE_2_ and PGD_2_ were comparatively analyzed in URB937-treated mice (URB937/KA) vs. combined, PEA- and URB937-treated (PEA+URB937/KA), and acute PEA-treated (PEA/KA) mice at 20, 60, 120, 180 min post KA injection, respectively. Error bars indicate SEM and asterisks in the figures indicate significant differences, *P < 0.05, **P < 0.01, ***P < 0.001.

In plasma, URB937-induced blockade of the peripheral FAAH rendered significantly increased AEA and PEA levels over the entire 180 min time span compared to PEA treatment only (Figure 6). Interestingly, the potency of URB937 in increasing AEA levels (Figure 6) was about two fold less compared to URB597 (Figure 5), but about two fold higher in increasing the PEA levels (Figures 5, 6). Thus, our data suggest that URB937 is more effective in inhibiting PEA degradation than URB597, but URB597 is more potent than URB937 in blocking AEA degradation (Figures 5, 6).

Plasma levels of 2-AG were significantly decreased upon URB937 administration and in co-administration with PEA, which indicates a similar 2-AG modulation as by URB597 and URB597/PEA treatments. PGE_2_ is maintained in the late-seizure phase (120–180 min) at higher levels upon URB937 and URB937/PEA treatment compared to PEA treatment (Figure 6).

Interestingly, the URB937 treatment had similar effects as the PEA treatment on the hippocampal response of AEA, 2-AG, AA, PGE_2_ and PGD_2_ to KA-excitotoxic stimulus (Figure 6). This effect is likely modulated by the high levels of endogenous PEA in hippocampus and plasma resulting from URB937 treatment (Figure 6, see “Discussion” section). In hippocampus, upon URB937 and URB937/PEA treatment the only significant changes compared to PEA treatment were that of PEA elevation over the entire time course and of PGE_2_ at the late phase (180 min; Figure 6).

Conclusively, the peripheral effects of URB937 on the AEA levels are less effective compared to PEA treatment to achieve a sustained anticonvulsive effect, and this concurs with significantly stronger epileptic seizure episodes compared to PEA treatment at acute and late-acute seizure phase (Figure 6). This also indicates that pharmacological modulation of peripheral FAAH tone only is not sufficient to sustain anticonvulsive effects.

## Discussion

The hippocampal response to the KA-induced excitotoxicity is an immediate (at 20 min) increase of AEA and AA, and AA-derived inflammatory markers PGE_2_ and PGD_2_, which happens prior to the occurrence of the spontaneous seizures (Figure 2). It is well established that AEA acts as an endogenous neuroprotectant, and its immediate increase at 20 min post KA injection functions as an “innate” hippocampal neuroprotective response to KA-induced excitotoxicity (Marsicano et al., 2003). The upregulation of AEA and AA-derived prostaglandins at the early onset of seizures and the reversal to basal levels by the time of acute seizure occurrence indicates a potential AEA-mediated activation of COX-2 as an autoprotective inflammatory mechanism to the KA-induced neurotoxicity. Conversely, the levels of AEA and AA significantly drop in plasma at early seizure onset (20 min; Figure 2), underscoring an involvement of AEA-mediated COX-2 activation across brain-periphery axis in response to KA-induced seizures. Moreover, the downregulation of AEA and AA in periphery up to the acute seizure state, followed by upregulation at late phase (180 min; Figure 2) supports the notion that the AEA-mediated COX-2 activation occurs during the seizure onset and progression. This prompts the hypothesis that AEA, through activation of COX-2 (Kozak et al., 2002), acts as one of the mediators of an autoprotective inflammatory mechanism triggered in response to KA-induced excitotoxic stimuli. The onset of peripheral inflammation occurs at the acute seizure state (at 60 min) shown by the elevation of PGE_2_ and progresses significantly through the elevation of the PGD_2_ as well after acute seizure state (120–180 min) with concomitant increase of AEA-AA, and regulation to basal level of the 2-AG likely through monoacylglycerol lipase (MAGL) digestion to AA production (Figure 2). Moreover, it appears that the acute seizure phase, e.g., 60 min, represents a turn-over time point in the dynamic of the eCBs and eiCs level changes: upregulation in hippocampus is accompanied by downregulation in periphery before the acute phase, and normalization to basal in hippocampus is followed by upregulation in periphery after the acute phase (Figure 2). It follows then that the seizure progression after the acute phase (60 min; Figures 1A,B) is correlated with an increase of inflammation in periphery, but not in the brain (Figure 2), e.g., hippocampus, suggesting peripheral inflammation as a possible contributing factor to seizure progression. Certainly, using the acute epilepsy mouse model with systemically injected KA we cannot conclude that the peripheral inflammation is a consequence of brain inflammation due to seizures or of the peripheral KA toxicity itself. Prospective studies on mouse model with intracerebral KA administration and in pilocarpine model will be instrumental to elucidate this aspect and its clinical relevance for patients with TLE. Nevertheless, irrespective of the origin of this peripheral inflammation, after acute epileptic state (60 min) increased seizure activity correlates with significant increase in plasma pro-inflammatory markers, which are both attenuated by PEA treatment (Figures 2, 5). Further elucidation and validation of these findings to draw reliable conclusions for TLE patients is certainly necessary.

Intraperitoneal administration of PEA completely restores to basal the up-regulation of AEA-AA-prostaglandins (PGs), which occurs in hippocampus in early response to KA-induced excitotoxicity (Figure 2). This PEA-regulation of the eCB and eiC levels in the hippocampus at the early stage (20 min) of KA-induced excitotoxicity, but prior to onset of seizures, leads to a significant decrease of seizure intensity in acute phase upon PEA treatment, which is even more prominent after the acute phase (60–180 min; Figure 1). The fact that PEA treatment reverses the increase of endogenous neuroprotective AEA, but with beneficial consequences for alleviation of seizure intensity, implies that the anti-inflammatory and neuroprotective actions of PEA *per se* alleviate the necessity of an “innate” brain inflammation response by AEA under excitotoxic stimuli and hence, supports the role of AEA in the brain inflammatory auto-defense mechanism (see above discussion). It remains though unclear how the intraperitoneal administered PEA affects the brain. Our data show that upon PEA treatment, the PEA levels in hippocampus rise significantly prior to and at acute seizure state (Figure 2), which might originate from an immediate uptake of PEA in the brain, and/or an increase of endogenous hippocampal PEA. However, the progressive anticonvulsant (Figure 1) and neuroprotective effects of PEA treatment (Supplementary Figures S4, S5) after acute seizure are not associated with an increased level of hippocampal PEA, but with a consistently high level of peripheral PEA, a decrease of peripheral inflammation, and regulation of peripheral eCBs (Figure 2). Accordingly, our data suggest that exogenous PEA exerts its neuroprotective, anticonvulsant and anti-inflammatory action across the brain-periphery axis. The exact mechanism of PEA action remains to be elucidated in further studies. Nevertheless, emerging evidence supports the notion that the neuroprotective and anti-inflammatory effect of PEA occurs through complex mechanisms involving interactions between microglial and non-neuronal cells of immune origin such as mast cells in the CNS, and with mast cells and macrophages in the periphery (Skaper et al., 2013; Herrera et al., 2016; Guida et al., 2017). In periphery, PEA completely reverses the upregulation of eCBs and eiCs, e.g., 2-AG and PGE_2_ in acute phase and maintains low the level of AEA-AA till acute phase, likely in order to tune down the rise of KA-derived peripheral inflammation by restricting the AA pool for PGs synthesis. Indeed, in the second phase of epilepsy (60–180 min), the upregulation of PGs is decreased and in turn, the levels of AEA and AA are completely normalized (Figure 2). Thus, our data indicate that the therapeutic effect of PEA relies in part on a modulation of eCB and eiC levels across the periphery-brain axis.

It has been suggested (Skaper and Facci, 2012; Skaper et al., 2014) that pre-administration of PEA prepares the body in a state of “hypervigilance” in the face of potential imminent negative stimuli by effectively modulating peripheral-brain immune system. We also administered PEA in control animals to understand how PEA modulates the eCBs and eiCs in both hippocampus and periphery prior to, or in the absence of, excitotoxic stimuli. Indeed, PEA significantly tunes down the peripheral AA pool and its precursor 2-AG for production of inflammatory markers, and subsequently of PGE_2_ and PGD_2_ (Supplementary Figure S3), which could represent a mechanism through which PEA prepares the body in the state of so-called “hypervigilance” to exert its anti-inflammatory properties. In hippocampus of PEA-treated controls, a massive increase of PEA levels occurs and is maintained over the entire time course in the absence of excitotoxic stimuli, accompanied by a slight increase of AA, yet no effect on AEA or inflammation markers PGE_2_ and PGD_2_ (Supplementary Figure S4). Intriguingly, exogenous administration of PEA in controls significantly elevates the plasma AEA level and steadily keeps it so over the entire time course, likely serving as a competitive substrate to FAAH (Supplementary Figure S3). Yet, under KA-induced cytotoxicity, the significant drop of AEA in plasma is not reversed by PEA through competing substrate despite availability of a high PEA source in plasma (Figure 2). Similarly, exogenous PEA changes the AEA, 2-AG and inflammation markers in the hippocampus only in the presence of KA-excitotoxic stimuli (Supplementary Figures S2, S4). This clearly indicates a PEA- mediated regulation of the peripheral and hippocampal AEA level that takes place specifically with an underlying pathological condition. Future mechanistic studies of the PEA antiepileptic role will be designed considering the intriguing role of PEA in modulation of peripheral and hippocampal AEA, and of periphery-brain cross-talk involved in antiepileptic effects.

Exogenous PEA administration has similar anticonvulsant properties (Figure 1) as those obtained by elevation of endogenous levels of AEA and PEA by URB597, or in combination (Figure 4A), despite the different mechanisms of actions (Figures 2, 5). Indeed, combinatorial administration of FAAH inhibitor with PEA does not substantially produce cumulative therapeutic effect in seizure alleviation (Figure 4), suggesting that PEA acts through a distinct mechanism, which is independent from any potential alteration of FAAH functionality in seizure. Specific increase of the peripheral PEA and AEA (Figure 6) by the selective peripheral FAAH inhibitor URB937 has the same effect on the brain levels of AEA, AA, and inflammatory markers at the early seizure phase (20–60 min) as the exogenous PEA administration (Figure 6). This effect, though surprising at first, does demonstrate that high pool of peripheral PEA produced by URB937-induced FAAH blockade (Figure 6) can modulate the brain AEA levels through a cross-talk between periphery and brain, and/or a massive uptake in the brain of URB937-induced peripheral PEA. The less effective anticonvulsant properties of the URB937 at acute- and late-acute seizure phase (Figure 4B) compared to PEA treatment (Figure 1A) might then stem from much higher peripheral and hippocampal inflammation, accompanied by a lower increase of plasma AEA, and complete lack of AEA increase in hippocampus (Figures 5, 6). Accordingly, FAAH blockade must necessarily occur in the brain in order to exert efficient anticonvulsive properties.

Evidently, these findings are limited and valid for this particular model of epilepsy and its restricted level of resemblance to human TLE. Future studies focused on rodent models better resembling TLE such as intra-hippocampal injected KA, and suitable models for seizure re-occurrence and/or chronic seizures, should be carried out to derive the long-term potential of PEA treatment and its clinical relevance for patients.

Subchronic administration of PEA was more effective in reducing seizure intensity (Figure 1) and had more significant effects on regulating the eCBs and eiCs compared to single dose injection of PEA (Supplementary Figures S1, S2), and it reduces the KA-induced neurodegeneration to almost 50% (Figure 3). Accordingly, these results highlight a promising potential of chronic PEA administration as an anticonvulsant therapy.

Moreover, PEA may represent a more beneficial therapeutic option than FAAH inhibitors, considering that it is a natural compound, does not show dose-dependent or long-term side effects, and its extensively reported direct anti-inflammatory properties. Although PEA modulates brain AEA levels in seizure, this effect is independent from a direct action on the eCB system whose ubiquitous and broad activation may be less beneficial in complex pathological conditions as epilepsy.

## Conclusions and Perspectives

Subchronic PEA treatment is an effective anticonvulsant therapy in KA-induced acute mouse model of epilepsy by significantly decreasing the seizure intensity at and after-acute seizure state, exerting significant neuroprotective effects in brain and downregulating the peripheral and hippocampal inflammatory responses to the excitotoxicity. These results highlight PEA as a promising candidate for chronic antiepileptic treatment. Prospective application of PEA treatment on other models resembling TLE in humans will help elucidating its applicability and potential beyond the current epilepsy model. To our knowledge, this is the first study that reveals time-point specific PEA-mediated antiepileptic effects, as well as eCB and eiC dynamics in KA-mouse model of epilepsy. Moreover, plasma lipids are shown to serve as viable candidates for follow-up drug monitoring, hence facilitating the quest of AED-therapies. The positive effects of PEA pre-treatment revealed here highlight its potential applicability in translational human studies that aim at establishing novel antiepileptic pharmaco-therapeutic strategies.

## Author Contributions

JMP performed experiments, assessed, prepared the data. SL co-performed animal experiments. RL co-performed lipidomic experiments. FR co-performed statistical analysis. EL guided pharmacological experiments, interpreted pharmacological data. BL assessed and interpreted data. LB designed and coordinated the study, interpreted data and wrote the article. All authors revised the manuscript.

## Conflict of Interest Statement

The authors declare that the research was conducted in the absence of any commercial or financial relationships that could be construed as a potential conflict of interest.
