# Supplementary material for: Antiepileptogenic Effect of Subchronic Palmitoylethanolamide Treatment in a Mouse Model of Acute Epilepsy
Source: Front Mol Neurosci. 2018 Mar 14;11:67. doi: 10.3389/fnmol.2018.00067 (PMC5861196; doi:10.3389/fnmol.2018.00067)
Supplement: Supplementary file 1 [file Data_Sheet_1.pdf]

Supplementary Material

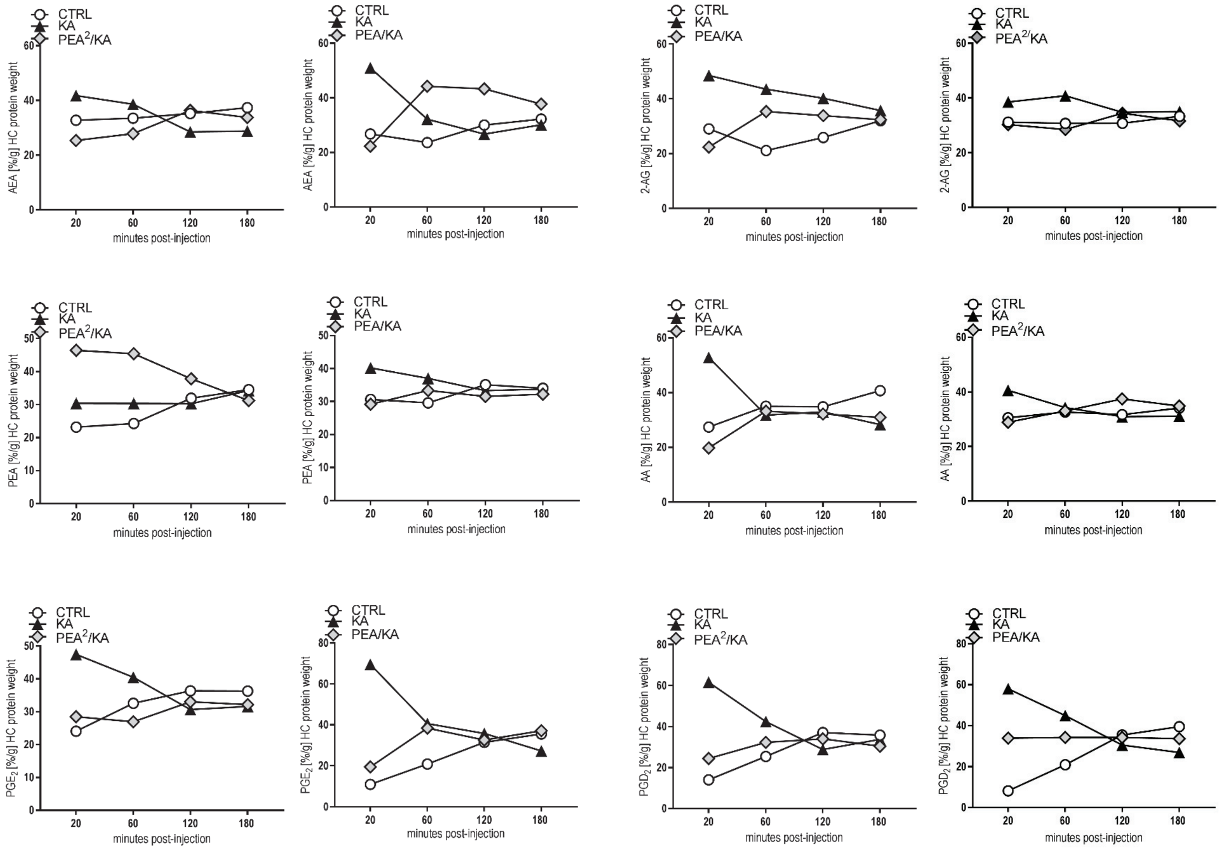

Suppl. Figure 1. Effect of acute and subchronic PEA-treatment on KA-induced epileptic seizures on hippocampal lipid (AEA, 2-AG, AA, PEA, PGE_2 and PGD_2) levels compared to vehicle injected controls over time course (20, 60, 120 and 180 min) post KA/vehicle-injection. Lipid levels are given in percentage, whereby the sum of average lipid levels of all three groups, at every time point was calculated and set to 100% and then percentages for every lipid level per time point and group were calculated. KA - kainic acid injected mice; PEA/KA - acute PEA-treated KA-injected mice; PEA^2/KA - subchronic PEA-treated KA-injected mice. Subchronic PEA-treatment modulates the lipid levels to an extent (%) closer to those of vehicle-injected group compared to acute PEA-treatment at the distinct time points.

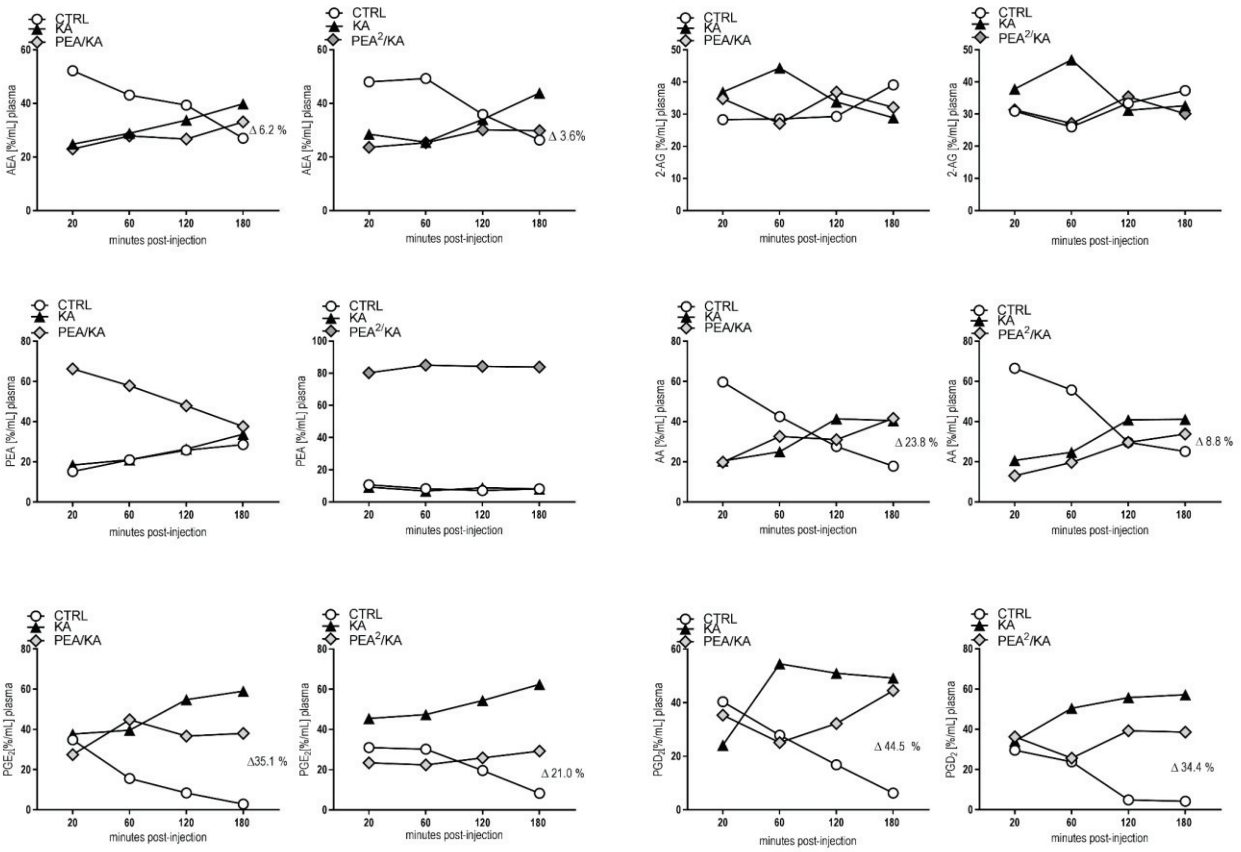

Suppl. Figure 2 Effect of acute and subchronic PEA-treatment on KA-induced epileptic seizures on plasma lipid (AEA, 2-AG, AA, PEA, PGE_2 and PGD_2) levels compared to vehicle-injected controls over time course (20, 60, 120 and 180 min) post KA/vehicle-injection. Lipid levels are given in percentage, whereby the sum of average lipid levels of all three groups, at every time point was calculated and set to 100% and then percentages for every lipid level per time point and group were calculated. KA - kainic acid injected mice; PEA/KA - acute PEA-treated KA-injected mice; PEA^2 /KA - subchronic PEA-treated KA-injected mice. Subchronic PEA-treatment modulates the lipid levels to an extent (%) closer to those of vehicle-injected group compared to acute PEA-treatment at the distinct time points. Δ % depicts the variation in % of the lipid levels upon PEA-treatment from the vehicle-injected. The Δ % values depicted for AEA, AA, PGE_2, PGD_2 at 180 min are all superior for subchronic PEA-treatment compared to acute PEA-treatment.

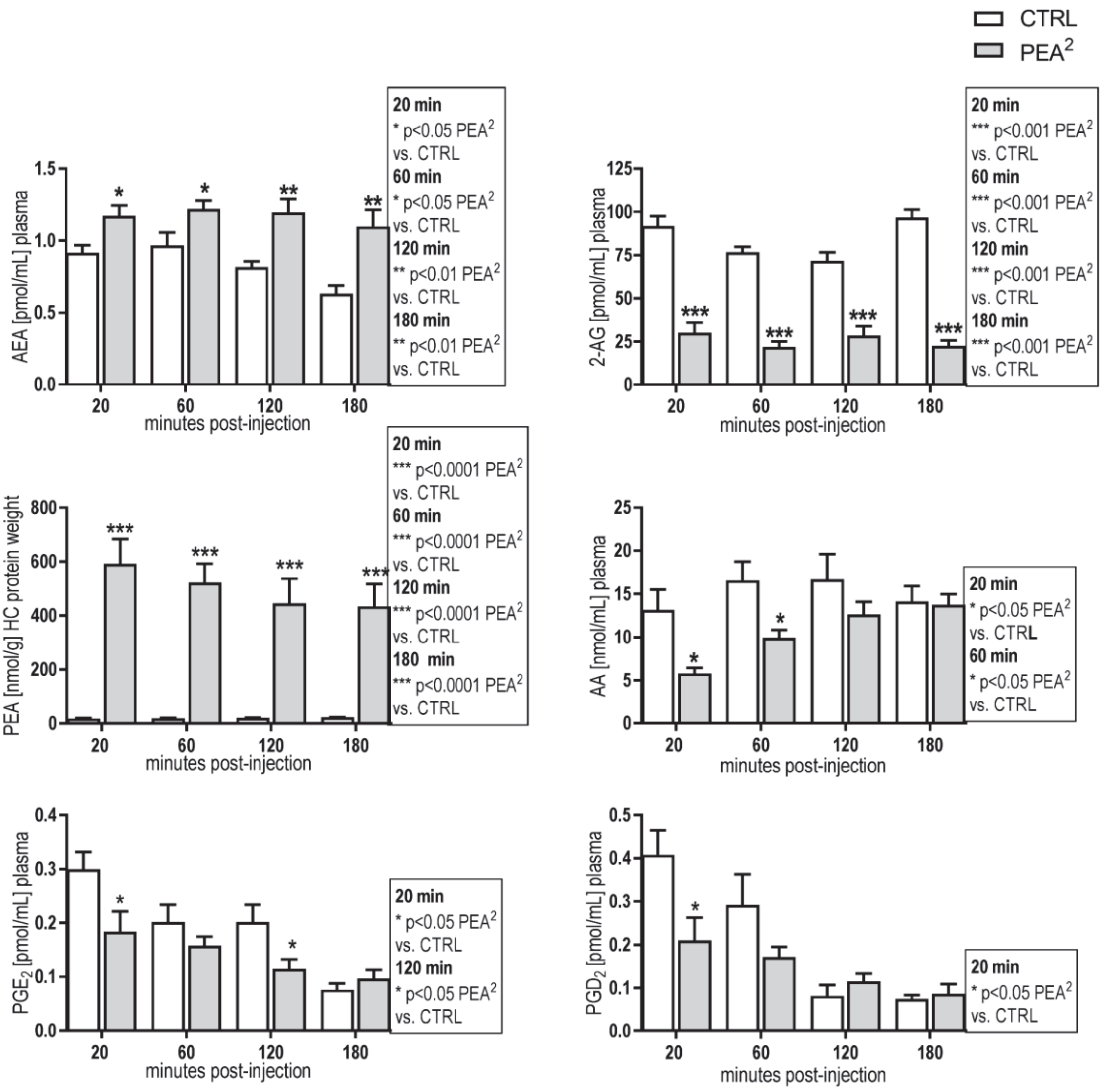

Suppl. Figure 3. Plasma lipid (AEA, 2-AG, PEA, AA, PGE_2 and PGD_2) level changes of healthy controls upon subchronic administration of PEA (PEA^2) vs. vehicle-injected control mice. Error bars indicate SEM and asterisks in the figures indicate significant differences, *P < 0.05, **P < 0.01, ***P < 0.001.

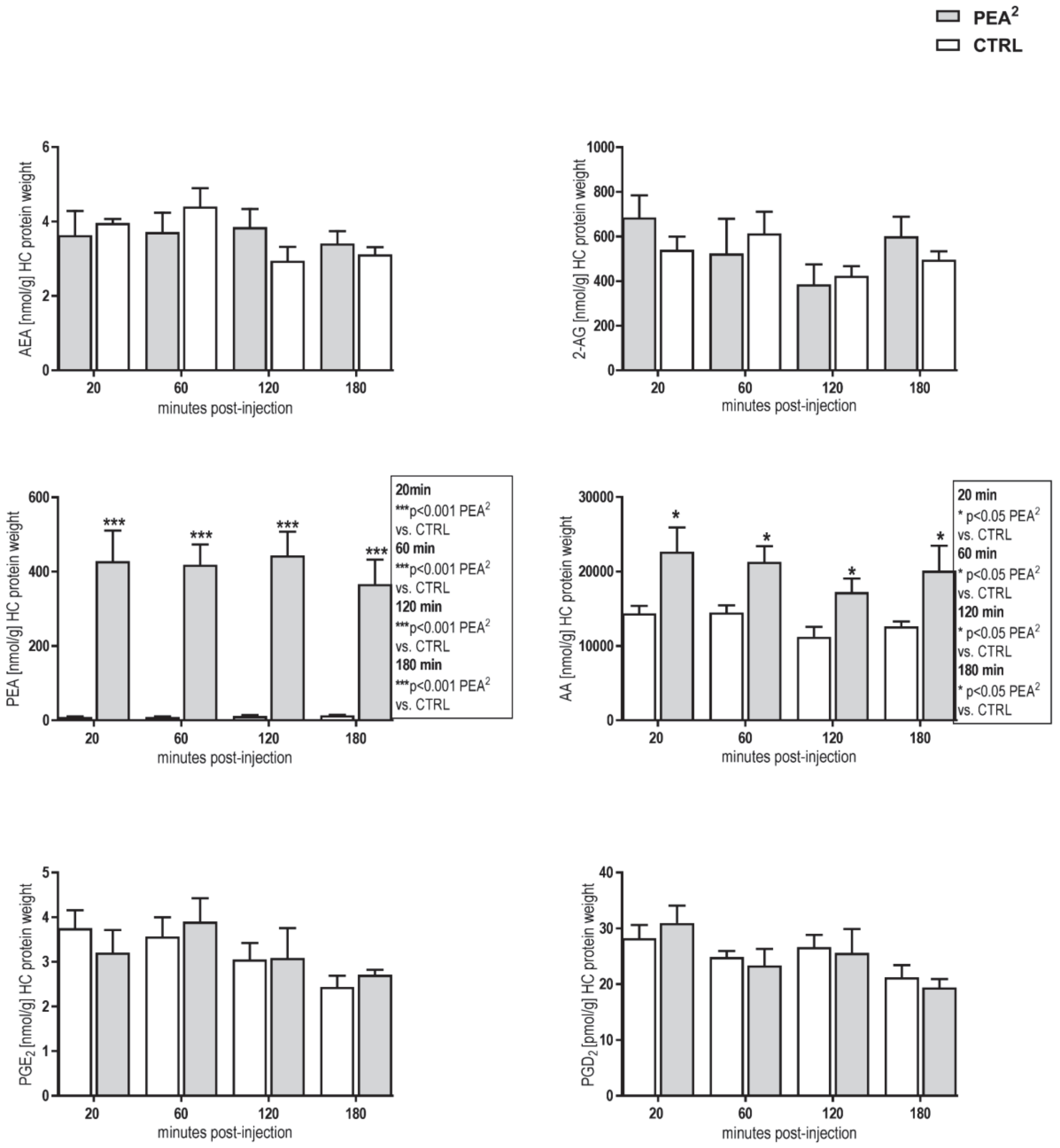

Suppl. Figure 4. Hippocampal lipid (AEA, 2-AG, PEA, AA, PGE_2 and PGD_2) level changes of healthy controls upon subchronic administration of PEA (PEA^2) vs. vehicle-injected control mice. Error bars indicate SEM and asterisks in the figures indicate significant differences, $*P < 0.05$, $***P < 0.001$.

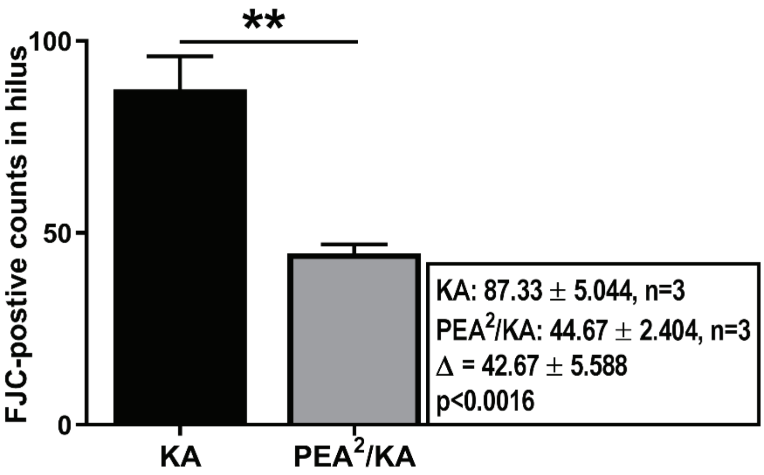

Suppl. Figure 5. Semi-quantitative comparison of positive FJC signals in the hilus region from PEA-subchronically-treated (PEA^2/KA) vs. untreated (KA) epileptic mice. FJC signal were manually counted in three subsequent coronal brain sections distanced by 200 µm. Unpaired t-test revealed significant reduction ($p < 0.0016$) of FJC-sensitive neurodegeneration in ROI after subchronic PEA-treatment. Error bars indicate SEM and asterisks in the figures indicate significant differences, **$P < 0.001$.
